# A CNN-Based Framework for Predicting Public Emotion and Multi-Level Behaviors Based on Network Public Opinion

**DOI:** 10.3389/fpsyg.2022.909439

**Published:** 2022-06-23

**Authors:** Hangfeng Lin, Naiqing Bu

**Affiliations:** ^1^School of Political Science and Public Administration, East China University of Political Science and Law, Shanghai, China; ^2^School of Sociology, Sanya University, Sanya, China

**Keywords:** network public opinion analysis, mental health of netizens, emotional tendency prediction, convolutional neural network, TF-IDF, multi-level government behaviors

## Abstract

Analysis of network public opinion can help to effectively predict the public emotion and the multi-level government behaviors. Due to the massive and multidimensional characteristics of network public opinion data, the in-depth value mining of public opinion is one of the research bottlenecks. Based on Term Frequency-Inverse Document Frequency (TF-IDF) and deep learning technologies, this paper proposes an advanced TF-IDF mechanism, namely TF-IDF-COR, to extract text feature representations of public opinions and develops a CNN-based prediction model to predict the tendency of publics' emotion and mental health. The proposed method can accurately judge the emotional tendency of network users. The main contribution of this paper is as follows: (1) based on the advantages of TF-IDF mechanism, we propose a TF-IDF-COR mechanism, which integrates the correlation coefficient of word embeddings to TF-IDF. (2) To make the extracted feature semantic information more comprehensive, CNN and TF-IDF-COR are combined to form an effective COR-CNN model for emotion and mental health prediction. Finally, experiments on Sina-Weibo and Twitter opinion data sets show that the improved TF-IDF-COR and the COR-CNN model have better classification performance than traditional classification models. In the experiment, we compare the proposed COR-CNN with support vector machine, k-nearest neighbors, and convolutional neural network in terms of accuracy and F1 score. Experiment results show that COR-CNN performs much better than the three baseline models.

## Introduction

In the new media era, publics tend to express their views on social events through the Internet. Yong people are extremely active in the network. Their enthusiasm and desire to participate in social life and the expression of subject consciousness are becoming stronger and stronger. They are used to overseeing the “voice box” of the network and have the appeal of expressing their own opinions and interests on social, cultural, economic, and other issues. They take the new media as a way and means to pursue their own value and express their individual consciousness. It is necessary to do intelligent analysis of the content, characteristics, and problems of young people's online public opinion in the new media era. We can improve the collection channels of young people's online public opinion information to achieve this.

Due to the massive and multidimensional characteristics of network public opinion data, the in-depth value mining of public opinion data has always been one of the bottlenecks research areas. In recent years, the rise and practicability of artificial intelligence technology has provided a new means and path for us to realize the automation, intelligence, and accuracy of network public opinion analysis. Therefore, some researchers have also made useful explorations. For example, some researchers used wavelet analysis to decompose the development process of public opinion (Gao et al., 2021), and then applied artificial neural network to model and predict the trend of public opinion (Punjabi et al., 2019). In addition, neural network simulation is adopted to simulate the development process of public opinion (Jelodar et al., 2020). Gray prediction and pattern recognition are employed to predict the trend of public opinion (Eagly et al., 2020).

The analysis and prediction of public opinion data is the most critical step in the network public opinion analysis technology. By designing appropriate algorithms to analyze the public opinion data, we can explore the hot topics, evaluate their communication impact and public opinion level, and adopt reasonable means to guide and control public opinion. In terms of public opinion analysis, the commonly used technical means include Bayesian classifier, support vector machine (SVM), random depth, and neural networks (De Gelder, 2010). Public opinion prediction is an important step after data analysis. This process is mainly to provide important reference for public opinion monitoring and public opinion early warning and help formulate relevant response measures for government departments and enterprises at all levels. At present, the research on network public opinion prediction is relatively extensive, and there are many methods to use (Eagly et al., 2020). Public opinion information mostly comes from short text comment information. Its text is separated from the written language, the structure becomes more concise and lack of standardization, which often makes it difficult to extract text features. Traditional emotion analysis methods often rely on emotion dictionary and feature extraction. With the continuous updating and iteration of Internet culture and data volume, many manuals updating of emotion dictionary is required, otherwise semantic features will be lost, and classification will be inaccurate.

Based on the analysis of TF-IDF (Term Frequency-Inverse Document Frequency) and deep learning technologies, this paper proposes a TF-IDF-COR (Term Frequency-Inverse Document Frequency-Correlation) mechanism to extract text representations of public opinions, and a Convolutional neural network (CNN)-based prediction model (Pavlova, 2017) to predict the risk of publics' mental health. The proposed method can accurately judge the emotional tendency of network users. The main work of this paper are as follows: (1) When there are TF (word frequency) and IDF (inverse document frequency), we multiply the two words to get the TF-IDF value of a word. The larger the TF-IDF of a word in the article, the higher the importance of the word in the article. Therefore, by calculating the TF-IDF of each word in the article, the top words are the keywords of the article. Based on the advantages of TF-IDF mechanism, we propose a TF-IDF-COR mechanism, which integrates the coefficients of word embeddings to TF-IDF. (2) CNN can better extract the local features of the text. To make the extracted feature semantic information more comprehensive, the two are combined to form a COR-CNN model. The COR-CNN model with the optimal parameters is obtained by comparing several groups of model parameters, which improves the classification performance compared with the traditional CNN models. Finally, experiments on Sina-Weibo and Twitter data set (Rodríguez et al., 2020) show that the improved TF-IDF-COR and the COR-CNN model has better classification performance than traditional classification models. In the experiment, we compare the proposed COR-CNN with SVM, KNN (K-Nearest Neighbor) and CNN models in terms of accuracy, recall and F1 score (Kobylińska and Kusev, 2019). Experiment results show that COR-CNN performs much better than the three baseline models. The main contribution of this work is as follows:

1. We propose a new feature extraction and representation mechanism, called TF-IDF-COR. It integrates the coefficients of word embeddings to TF-IDF to find top words of the keywords of an article more efficiently.
2. We propose a CNN-based model, called COR-CNN to extract feature semantic information by considering the coefficients of word embeddings.
3. We conduct comprehensive experiments based on Sina-Weibo and Twitter data set for evaluate the proposed TF-IDF-COR and COR-CNN. They are compared with three classical machine learning algorithms. The experiment proves that the proposed methods are effective and efficient.

The structure of the remaining paper is: Section Related work introduces the related work about the machine learning algorithms for risk analysis of network public opinions and the machine learning algorithms for mental health evaluation and prediction. Section Text Representation Learning and Risk Prediction of Network Public Opinions introduces the proposed CNN-based framework for emotion and multi-level behavior prediction based on the network public opinions. Section Experiment Setting and Result Analysis shows the experiment design and result comparison with baseline models. Section Conclusion presents the paper conclusion and the future work.

## Related Work

Lorenz-Spreen et al. (2020) proposed two classes of behavioral interventions-nudging and boosting-that enlist these cues to redesign online public opinion to promote deliberate cognition and autonomous choice. Pickett (2019) reviewed evidence for the effects of public opinion on court decision-making, capital punishment policy and use, correctional expenditures, and incarceration rates. D'Andrea et al. (2019) automatically inferred trends in the public opinion regarding the stance toward the vaccination topic. It enables the detection of significant opinion shifts. These opinion shifts can be possibly explained with the occurrence of specific social context-related events. This study (Han et al., 2020) uses random forest algorithm explored public opinion in the early stages of COVID-19 in China by analyzing Sina-Weibo texts in terms of space, time, and content. In controlling the crisis, accurate response countermeasures should be formulated following public help demands. The study (Jia and Chen, 2020) applies emotional analysis is to the evolution analysis of network public opinion, and the change of network public opinion characteristics with time series is obtained, which can get the change of emotional characteristics of public opinion participants with time series.

The research work (Srividya et al., 2018) proposes to apply various machine learning algorithms such as support vector machines, decision trees, naïve bayes classifier, K-nearest neighbor classifier and logistic regression to identify state of mental health in a target group. Shatte et al. (2019) point out there is significant room for the application of machine learning to other areas of psychology and mental health. The challenges of using machine learning techniques are discussed. The opportunities to improve and advance the machine learning algorithms for text analysis is also analyzed. The work (Wilkinson et al., 2017) is a study of postnatal depression in women using a decision tree model, postnatal depression screening and treatment is a cost-effective intervention that should be considered as part of routine postnatal care. The work (Alonso et al., 2018) applies data mining techniques to mental health disorders such as dementia, schizophrenia, and depression. Such techniques can be of great help in clinical decision-making, diagnostic prediction and improving the quality of life of patients. This paper (Garcia-Ceja et al., 2018) surveys recent research works in mental health monitoring systems (MHMS) using sensor data and machine learning.

## Text Representation Learning and Risk Prediction of Network Public Opinions

In this section we propose a CNN-based framework, called COR-CNN for network public opinions classification. At first, we need to extract text summaries from public opinions and news. Then we use the external corpus to train the word vector model, convert the text representations extracted from the opinions into a vectorized representation based on the word vector, and then concatenate the vectorized representation of the sentence into a vectorized representation of the entire text. Finally, the convolutional neural network is trained, and the trained network model is used to predict the emotion of netizens.

### Learning Text Representations in Public Opinions

Compared with the data in the form of online comments, news texts are generally longer. And the difference between the lengths of the texts is also large, so the texts must be processed before use. Sentences can more accurately grasp the semantics of text than words, so we solve this problem by extracting text summaries of texts. For text classification, the traditional TF-IDF algorithm will lose some key classification criteria. Therefore, we will design an enhanced TF-IDF algorithm to solve this problem. Then, the top K sentences with the highest scores are selected as the text summary of the text, which reduces the data dimension and eliminates noises.

#### TF-IDF-COR Algorithm

TF-IDF considers the text set as a whole, and its IDF part does not consider the inter-class distribution information of feature items. If the entry *t*_*a*_ has a high frequency of occurrence in a certain category *c*_*a*_, which leads to the occurrence of the entry *t*_*a*_ in more texts. Although the entry *t*_*a*_ appears less in other categories, the weight calculated according to the IDF algorithm will be too small. The entry *t*_*a*_ will be mistaken for an entry with poor ability to distinguish between categories. Obviously, this is not in line with the actual situation. Words that only appear frequently in a certain category or categories are the most one or several categories of iconic words have high information value for text classification, so they should be given high weights. Correspondingly, if the entry *t*_*a*_ appears only in a small amount of text. The frequency of occurrence in each class is relatively uniform. This kind of unimportant word will be given very high weight by the IDF algorithm, so it is not in line with the actual situation. When the distribution of a word under different topic news is quite different, it means that the word has a strong representativeness for a certain category or categories of news and can be used as a key basis for classification. The above-mentioned defects are because the traditional TF-IDF algorithm does not consider the distribution information of words between classes, which obviously loses part of the accuracy when performing text classification. Therefore, we measure the inter-class distribution information of words by adding an inter-class correlation coefficient (Wang et al., 2012).

#### Learn Text Representations

For the task of text representation extraction, the goal is to extract the most important set of sentences in the text. First, the weights of the terms are calculated by an improved TF-IDF algorithm. Suppose D is a text collection. For any word *w*_*a*_ in the text *D*_*b*_, the word frequency is expressed by Equation 1.


(1) $$\begin{array}{l} tf_{ab}=\frac{n_{ab}}{\sum_{k}n_{kb}} \end{array}$$

where *n*_*ab*_ represents the number of occurrences of the word *w*_*a*_ in the text *D*_*b*_, and m represents the length of the dictionary.

The inverse document frequency is expressed by Equation 2.


(2) $$\begin{array}{l} idf_{a}=\log\frac{|D|}{1+|\left\{D_{b}:t_{a}\in D_{b}\right\}|} \end{array}$$

The inter-class dispersion of words is expressed by Equation 3.


(3) $$\begin{array}{l} ICD_{a}=\frac{\sqrt{\frac{1}{c-1}\sum_{r=1}^{c}{\left(tf_{ak}\left(w_{ar}\right)-\bar{tf_{ak}\left(w_{ar}\right)}\right)}^{2}}}{tf_{ar}\left(w_{ar}\right)} \end{array}$$

In the formula: $\bar{tf_{ar}}=\frac{1}{c}\overset{n}{\underset{a=1}{\sum }}tf_{ar}\left(w_{ar}\right)$, where *tf*_*ar*_ represents the number of occurrences of the word *w*_*a*_ in the L class, and c represents the number of text classes.

The product of Equations 1–3 is the TF-IDF-COR value of the corresponding term.

After obtaining the weights of all terms, the importance of the sentence is represented by the accumulation of the weights of the terms in the sentence. In addition, considering the different lengths of sentences, the results need to be processed accordingly to prevent the selected results from being biased toward long sentences. For a given sentence T, it can be represented by the terms contained in T: *T* = (*o*_1_, *o*_2_ ⋯ *o*_*n*_), the importance of T is defined in Equation 4.


(4) $$\begin{array}{l} I\left(T\right)=\frac{\sum_{a=1}^{m}tf_{ab}^{*}idf_{a}^{*}ICD_{a}}{\log\left(|T|+1\right)} \end{array}$$

where tf _ab_ represents the terms of occurrences of the word w _a_ in the sentence T _b_, and ICD _a_ represents the inter-class dispersion of words represents the length of the dictionary (see Equation 3).

Finally, to obtain text representations, all sentences of the text are ranked. The top K sentences for scoring are selected as the representation of the current text.

#### Determine the Embedding Distribution of Words in Public Opinions

To describe the semantic embedding distribution of words in each paper, an abstract distributed convolutional layer is designed. The text representation I(T) of the words in the sub-word is used as input. A cross entropy function is used to generate the embedding distributed representation of each word. Formally, using semantic information provided by *I*_*a*_, the expression order of abstract semantic field distribution of sub-words, K = {*k*_1_, *k*_2_, …, *k*_*N*_}, can be calculated by Equations 5–7.


(5) $$\begin{array}{l} g_{a}=W_{r}I_{a}+b_{r} \end{array}$$

(6) $$\begin{array}{l} r_{a}=\text{softmax}\left(g_{a}\right) \end{array}$$

(7) $$\begin{array}{l} r_{a,b}=\;\frac{e^{m_{ab}}}{\sum_{k=1}^{d_{2}}e^{m_{i,k}}} \end{array}$$

where, *W*_*r*_ and *b*_*r*_ are parameters, $W_{r}\in {\mathbb{R}}^{d_{3}\times d_{4}}$,*m*_*i*_,$b_{r}\in \;{\mathbb{R}}^{d_{3}}$.

There is naturally a gap inconsistency between sematic words and non-semantic words. Moreover, the greater the inconsistency between the semantic words and the semantic area of the text, the greater the possibility that the sematic words will be determined. Using the total distributed distance between semantic words and non-semantic words in the sub-word, the semantic area of the sub-word is not consistent. That is, the minimum area word embedding distribution distance between each semantic word and non-semantic words. The semantic area inconsistency of sematic embeddings is relatively high, and the goal is to maximize the semantic area inconsistency of sub-word. Choosing the minimum area distance can ensure that the local inconsistency between each pair of semantic words and non-semantic words is modeled correctly. The semantic field of the sentence T semantic area inconsistency SAI(T) is calculated by Equation 8.


(8) $$\begin{array}{l} SAI\left(T\right)=\overset{J}{\underset{j=1}{\sum }}\underset{i\geq u\geq U}{\min}\left\{Dista\left(r_{j}^{\left(m\right)},r_{u}^{\left(u\right)}\right)\right\} \end{array}$$

where, $r_{k}^{\left(met\right)}$, $r_{u}^{\left(un\right)}$ represent the semantic area of the embedding word *t*_*i*_ and the non-metaphorical word *t*_*u*_ in the sentence, respectively. $Dis\left(r_{j}^{\left(m\right)},r_{u}^{\left(u\right)}\right)$ expresses distance function of $r_{j}^{\left(m\right)}\;and\;r_{u}^{\left(u\right)}$. More than one distance measures exist in real life.

Distance 1: Kullback-Leibler dispersion distance is calculated by Equation 9.


(9) $$\begin{array}{l} D\left(r_{ki},r_{uj}\right)=\overset{d_{2}}{\underset{m=1}{\sum }}r_{k_{im}}^{*}\log\left(\frac{r_{i_{m}}}{r_{j_{m}}}\right) \end{array}$$

Distance 2: Euclidean distance is calculated by Equation 10.


(10) $$\begin{array}{l} D(r_{ki},r_{uj})=\sqrt{\sum_{m=1}^{d_{2}}{\left(r_{ki_{m}}-r_{j_{m}}\right)}^{2}} \end{array}$$

Distance 3: Korrigierter Kosinusabstand is calculated by Equation 11.


(11) $$\begin{array}{l} D\left(r_{i},r_{j}\right)=-\frac{\sum_{m=1}^{d_{2}}r_{i_{m}}r_{j_{m}}}{\sqrt{\sum_{m=1}^{d_{2}}{\left(r_{i_{m}}\right)}^{2}}\sqrt{\sum_{m=1}^{d_{2}}{\left(r_{j_{m}}\right)}^{2}}} \end{array}$$

Distance 4: Gaussian distance is calculated by Equation 12.


(12) $$\begin{array}{l} D(r_{ki},r_{uj})=\exp(-\frac{\sqrt{{\sum_{m=1}^{d_{2}}\left(r_{ki_{m}}-r_{j_{m}}\right)}^{2}}}{2\sigma^{2}}) \end{array}$$

### Tensorization Words

Tensorization words refers to the transformation of words in a language into digital representations that are easy for computers to process. The NNLM and the Log-Linear model are both outstanding representatives of using neural networks to obtain word vectors. The well-known Word2vec model is borrowed from these two and is a more concise and efficient word vector model. Word2vec technology is a key breakthrough in the application of deep learning technology in autologous language processing.

#### Word2vec and Skip Gram

Word2vec is a group of related models used to generate word vectors. These models are shallow and double-layer neural networks used for training to reconstruct linguistic word texts. The network is represented by words. The input words in adjacent positions need to be guessed. Under the assumption of word bag model in word2vec, the order of words is not important. After training, word2vec model can be used to map each word to a vector, which can be used to represent the relationship between words. This vector is the hidden layer of neural network.

Skip gram is a simple but very practical model. In natural language processing, the selection of corpus is a very important problem. At first, the corpus must be sufficient. On the one hand, the number of words in the dictionary should be large enough. On the other hand, it should include as many sentences reflecting the relationship between words as possible. For example, only when the sentence pattern of “fish swimming in the water” is as many as possible in the corpus, can the model learn the semantic and grammatical relationship in the sentence. This is the same as human learning natural language. If people repeat more times, they will imitate. Second, the corpus must be accurate. In other words, the selected corpus can correctly reflect the semantic and grammatical relationship of the language, which seems not difficult to achieve. For example, in Chinese, the corpus of people's daily is more accurate. However, more often, it is not the selection of corpus that raises concerns about accuracy, but the method of processing. In the n-ary model, due to the limitation of window size, the relationship between words beyond the scope of the window and the current word cannot be correctly reflected in the model. If the window size is simply expanded, it will increase the complexity of training. The skip gram model solves these problems.

After extracting the text representations from the text, the sentences are tensorized based on the word embedding vector model. The word2vec and corpus tool are used to train the model. For a sentence *T* = (*t*_1_, *t*_2_ ⋯ *t*_*n*_), for any term *M*_*i*_ = (*m*_1_, *m*_2_ ⋯ *m*_*k*_), where k represents the dimension of the word. The tensorized representation of statement T is *T* = (*t*_1_ ⊕ *t*_2_ ⊕ ⋯ ⊕ *t*_*n*_), where ⊕ is the concatenation operator. Therefore, sentence T is tensorized to a series of ordered vectors. Similarly, for each text *P* = (*T*_1_ ⊕ *T*_2_ ⊕ ⋯ ⊕ *T*_*l*_), where L represents the order of importance of sentence T. That is to say, *T* is the most important sentence of the text P. After converting the text into vector form, the vectorized data can be used to train the neural network.

#### A CNN Model for Word Semantic Analysis in Public Opinions

Convolutional neural networks are composed of convolutional layers, pooling layers, and fully connected layers. The convolutional layer extracts the features of the data through convolutional computation. The pooling layer selects the optimal features from the features provided by the convolutional layer, and then outputs them to the fully connected layer for processing. The input of the input layer is a matrix representing the text, k represents the dimension of the word vector, and m represents the number of word vectors contained in each data. The convolution layer involves the convolution kernel *k* ∈ *R*^*lj*^, l represents the size of the convolution window, and j is the convolution dimension, which is equal to the dimension of the word vector. In general, *T*_*i* : *i*+*h*_ represents the word *T*_*i*_, *T*_*i*+1_ ⋯ *T*_*i*+*h*_. The expression to generate a text feature is *F* = *f*(*t* • *T*_*i* : *i*+*h*+*a*_), where a is the bias and f is a non-linear function. Applying this convolution kernel to (*T*_1:*h*_, *T*_2:*h*+1_ ⋯ *T*_*N*−*h*+1:*N*_) generates a feature map *p* = (*p*_1_, *p*_2_ ⋯ *p*_*N*−*h*+1_). The pooling layer uses the maximum pooling method to sample the feature map, and only retains the most important features of each feature group: *p*_max_ = p_*i*_. The multiple feature vectors output by the pooling layer are spliced and input to the input of the fully connected layer. The loss function of this COR-CNN model is defined in Equation 13.


(13) $$\begin{array}{l} Loss=\frac{1}{L}\overset{L}{\underset{k=1}{\sum }}\overset{N_{k}}{\underset{i=1}{\sum }}\gamma_{y_{i}}{\left(1-\hat{y_{i}}\right)}^{\delta }\log\left(\hat{y_{i}}\right)-\mu H\left(T_{L}\right) \end{array}$$

where,


(14) $$\begin{array}{l} H(T)=\{\begin{array}{cc} \;SAI\;(S_{l}) & \;S_{l}\;is\;semantic\\ -SAI\;(S_{l})\; & S_{l}\;is\;Non\;semantic \end{array} \end{array}$$

## Experiment Setting and Result Analysis

### Experiment Settings

The data used in this experiment comes from the Twitter public opinion corpus (Pak and Paroubek, 2010) and Sina public opinion corpus (Xue et al., 2014). Some opinion data are selected, covering six categories of education, economy, health, military, tourism, and sports. For data preprocessing, a word segmentation tool is used to segment the dataset and remove stop words. After that, the text representations are extracted by using the TF-IDF-COR algorithm. If the text and sentences are too long or too short, they are truncated to a fixed length or filled with blank data. The word vector adopts the skip-gram model in word2vec and is trained using the Wikipedia Chinese and English corpus.

Experiments are designed to evaluate the performance of the sentence-level news classification scheme based on the convolutional neural network model. To evaluate the effect of the classification scheme, the accuracy, recall, and F1 score are used as matrices to measure the performance of the CNN model. To reflect the superiority of the model and verify the model, a group of experiments using the SVM model for text classification was set as a baseline group. In addition, to illustrate the advantages of using the TF-IDF-COR algorithm in the information extraction stage of this mechanism, a set of comparative experiments using the TF-IDF-COR are set up on the same data set.

### Experiment Result

Table 1 is the comparison results of the proposed mechanism with two traditional classification models KNN, SVM, Naïve Bayes (NB) and Long Short-tem Memory network (LSTM) in terms of precision (Precision), recall (Recall), and F1 score.

**Table 1:** Performance comparison of different classification models.

**Classification model**	**Precision**	**Recall**	**F1**
KNN	0.832	0.798	0.827
SVM	0.897	0.875	0.882
NB	0.8333	0.7838	0.8052
LSTM	0.9221	0.8986	0.9147
TF-IDF-COR	0.957	0.949	0.953

As we can see from Table 1, the opinion classification algorithm based on the proposed TF-IDF-COR and CNN model is more accurate and more stable than traditional KNN, SVM, NB, and LSTM. On the one hand, the convolutional neural network model can extract richer classification features by increasing the convolution kernel. On the other hand, it can also extract higher-level classification features by increasing the number of convolutional layers. To put it simply, convolutional neural networks can extract richer features horizontally and more levels of features vertically, which is incomparable to traditional machine learning models.

Table 2 shows the comparison results of the precision rate (Precision), recall rate (Recall), and F1 score of the CNN model by using the traditional TF-IDF and the TF-IDF-COR, respectively, when learning text representations.

**Table 2:** Influence of different representation learning mechanism on risk classification results.

	**Precision**		**Recall**		**F1**	
	**TF-IDF**	**TF-IDF-COR**	**TF-IDF**	**TF-IDF-COR**	**TF-IDF**	**TF-IDF-COR**
Sports	0.841	0.903	0.884	0.947	0.857	0.928
Health	0.910	0.956	0.924	0.987	0.911	0.978
Travel	0.876	0.939	0.839	0.926	0.845	0.935
Military	0.912	0.978	0.933	0.945	0.925	0.959
Education	0.903	0.929	0.892	0.917	0.884	0.902
Economy	0.942	0.983	0.926	0.976	0.933	0.980

From Table 2, we can see that compared with the traditional TF-IDF algorithm, the average accuracy of the TF-IDF-COR is higher. The accuracy of the categories is more balanced. The higher accuracy in the original mechanism is only slightly reduced in the new mechanism, and the lower accuracy in the original mechanism is greatly improved in the new mechanism. Therefore, by improving the TF-IDF algorithm to capture different distributions of word embeddings between classes, the performance of CNN in text classification can be further improved.

**Figure 1:**
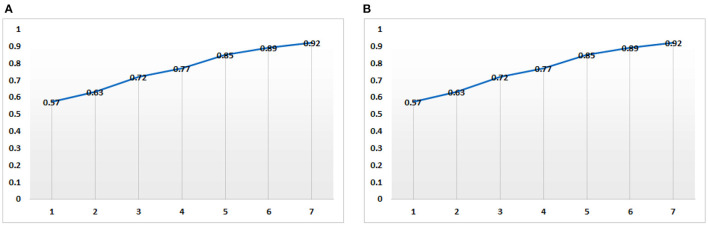
Change trend of mental disease risk prediction accuracy along the change of term volumes (KB) based on Twitter **(A)** and Sina **(B)** datasets.

As can be seen from Figure 1, with the change of term volumes (from 1 to 6 k terms), the risk prediction accuracy on both Twitter and Sina datasets are gradually increasing. With the accumulation of the public opinion data, the effect of predicting the public mental diseases based on the public's opinions will become better and better. On the other hand, it is also reflected that with the increase of the public's time online, the potential occurrence of public mental diseases in the future will become more and more clear.

## Conclusion

This paper proposed an advanced TF-IDF-COR mechanism to extract text representations of public opinions, and a CNN-based prediction model to predict the risk of publics' mental health. The proposed method can accurately judge the emotional tendency of network users and government behaviors. The proposed TF-IDF-COR mechanism integrates the correlation coefficients of word embeddings to TF-IDF. CNN and TF-IDF-COR are combined to form a COR-CNN model. Finally, experiments on Sina-Weibo and Twitter data sets prove that the improved TF-IDF-COR and the COR-CNN model have better classification performance than traditional classification models. In the experiment, we compare the proposed COR-CNN with SVM, KNN and CNN models in terms of accuracy and F1 score. Experiment results show that COR-CNN performs much better than the three baseline models. As we used a CNN model to extract features, which is time consuming. The time and space efficiency of the proposed methods should be future improved. In the future, we will take more lightweight models like ResNet to improve the CNN-based feature extraction model to improve its time and space efficiency.

## Data Availability Statement

The original contributions presented in the study are included in the article/supplementary material, further inquiries can be directed to the corresponding author.

## Author Contributions

HL designed the framework, collected data, and did the experiment. NB proof read the paper. Both authors contributed to the article and approved the submitted version.

## Conflict of Interest

The authors declare that the research was conducted in the absence of any commercial or financial relationships that could be construed as a potential conflict of interest.

## Publisher's Note

All claims expressed in this article are solely those of the authors and do not necessarily represent those of their affiliated organizations, or those of the publisher, the editors and the reviewers. Any product that may be evaluated in this article, or claim that may be made by its manufacturer, is not guaranteed or endorsed by the publisher.
